# Association between SARS-CoV-2 booster vaccination and hospitalisation and/or death due to COVID-19 in adults with immune-mediated inflammatory diseases: nested case–control study using linked primary-care, hospitalisation and mortality data from England

**DOI:** 10.1136/rmdopen-2026-006834

**Published:** 2026-07-03

**Authors:** Niraj S Kukreja, Georgina Nakafero, Abhishek Abhishek

**Affiliations:** 1 University of Southampton, Southampton, UK; 2 Academic Rheumatology, City Hospital Nottingham, University of Nottingham, Nottingham, UK; 3 NIHR Nottingham Biomedical Research Centre, Nottingham, Nottinghamshire, UK

**Keywords:** COVID-19, Autoimmune Diseases, DMARD

## Abstract

**Objectives:**

To assess the association between COVID-19 booster vaccination and hospitalisation and/or death due to COVID-19 among adults with immune-mediated inflammatory diseases (IMIDs).

**Methods:**

Adults with IMIDs prescribed steroid-sparing immune-suppressing drugs in Clinical Practice Research Datalink Aurum linked to hospitalisation and mortality records were included. Cases were hospitalised for or died due to COVID-19. Controls were risk-set matched to cases. Exposures were number of COVID-19 booster vaccinations between 16 September 2021 and 13 March 2023; receipt of autumn 2021, spring 2022, autumn 2022 boosters; and time since last booster. Multivariable logistic regression was used. Adjusted OR (aOR) and 95% CIs and adjusted vaccine effectiveness (1 − aORs × 100%) were calculated.

**Results:**

We included 2178 cases and 17 750 controls (mean age 65.5 years; 60.2% women). Hospitalisation and/or death due to COVID-19 was negatively associated with receipt of three (aOR (95% CI) 0.20 (0.15 to 0.28)), two (aOR (95% CI) 0.29 (0.23 to 0.36)) or one booster (aOR (95% CI) 0.56 (0.48 to 0.65), respectively, compared with no booster. The aOR (95% CI) across the booster cycles were: autumn 2021 (aOR (95% CI) 0.40 (0.32 to 0.50)), spring 2022 (aOR (95% CI) 0.52 (0.42 to 0.65)) and autumn 2022 (aOR (95% CI) 0.33 (0.25 to 0.43)). Hospitalisation and/or death due to COVID-19 was negatively associated with booster vaccination within 365 days compared with no booster vaccination. There was no statistically significant association for boosters received more than 365 days ago.

**Conclusions:**

COVID-19 booster vaccination was associated with fewer hospitalisation and/or death due to COVID-19 among adults with IMIDs. These findings support regular booster vaccination in this high-risk population.

**WHAT IS ALREADY KNOWN ON THIS TOPIC**

- People with immune-mediated inflammatory diseases (IMIDs) treated with immune-suppressing drugs are at an increased risk of hospitalisation and death due to COVID-19.
- The uptake of COVID-19 booster vaccination has reduced in this population. Lack of evidence about benefit from booster vaccinations has contributed to this decline.

**WHAT THIS STUDY ADDS**

- In adults with IMIDs prescribed immune-suppressing drugs, booster vaccinations against COVID-19 were associated with lower odds of hospitalisation or death due to COVID-19 with an adjusted OR of 0.56 for one, 0.29 for two and 0.20 for three booster vaccinations.
- Protection was observed during each booster campaign and was greatest shortly after booster vaccination, waning over time.

**HOW THIS STUDY MIGHT AFFECT RESEARCH, PRACTICE OR POLICY**

- The findings of this study support the need for regular booster vaccination in adults with IMIDs treated with immune-suppressing drugs and could be used to promote COVID-19 vaccination in this at-risk group.

## Introduction

 Immune-mediated inflammatory diseases (IMIDs) such as rheumatoid arthritis (RA), axial spondyloarthritis (AxSpA), systemic lupus erythematosus (SLE), inflammatory bowel disease (IBD), psoriasis and atopic dermatitis affect 6–7% of UK adults.^1^,^6^ They are treated with immune-suppressing drugs such as methotrexate. These medications blunt vaccine-induced immunity and increase susceptibility to severe COVID-19.^7 8^ People with IMIDs are at a significantly increased risk of hospitalisation and death due to COVID-19,^9^ with pooled OR of 1.73 and 1.92, respectively, in people with RA.^10^ The Joint Committee on Vaccination and Immunisation (JCVI) prioritised immunosuppressed individuals for primary vaccination and recommended 6 monthly boosters in the UK, called autumn and spring boosters.^11^,^14^ Annual vaccination against COVID-19 is recommended in the USA and whether 6 monthly boosters provide additional benefit is not known.^15^ Vaccine hesitancy, often driven by perceived lack of efficacy, safety concerns and fear of disease flare, remains a barrier to vaccine uptake in this population.^16^ Only 39% of booster-hesitant patients eventually received a vaccine dose.^17^

COVID-19 boosters augment vaccine-induced immunity, providing biological plausibility that increasing number of boosters received would be associated with a lower risk of hospitalisation and death due to COVID-19.^18^ We evaluated the association between hospitalisation and/or death due to COVID-19 and the number of booster vaccinations against COVID-19. We also explored the association between hospitalisation and/or death due to COVID-19 and individual COVID-19 booster vaccinations and examined the durability of protection.

## Methods

### Study design and data source

This was a nested case–control study. We used data from the Clinical Practice Research Datalink (CPRD) Aurum linked to Hospital Episode Statistics (HES) Admitted Patient Care and Office for National Statistics (ONS) databases. CPRD is a longitudinal database including anonymised electronic primary-care health records from the UK.^19^ It contains information about demographic details, comorbidities, lifestyle factors, test results, vaccinations, investigations and prescriptions. Linkage with the HES and ONS databases provides dates of hospitalisation and discharge diagnoses and dates and causes of death, respectively.

### Population

Adults (age≥18 years) registered in their current general practice (GP) surgery for ≥12 months on 16 September 2021 were eligible provided they had received ≥1 primary COVID-19 vaccine dose before this date and had a diagnosis of an IMID (RA, psoriatic arthritis, AxSpA, polymyalgia rheumatica, SLE, other connective-tissue disease, vasculitis, atopic dermatitis or IBD) recorded in primary care. They were required to have been prescribed an immune-suppressing drugs (methotrexate, azathioprine, 6-mercaptopurine, sulfasalazine, 5-aminosalicylate, mycophenolate, leflunomide, ciclosporin, tacrolimus or sirolimus) within 90 days before 16 September 2021. The 90-day window ensured that only patients currently prescribed an immune-suppressing drug and eligible for a COVID-19 booster vaccination were included. Using both diagnosis and prescription has 84% sensitivity and 86% specificity for ascertaining RA,^20^ and a primary care record of IBD has 92% accuracy in the CPRD.^21^ While validation studies have not been undertaken for all IMIDs, we anticipate a similarly high level of validity of the selected diseases.

### Follow-up

Started on 16 September 2021, that is, the start of COVID-19 booster roll-out in the UK and ended at the earliest date of: study end (13 March 2023), transferring out of the General Practice (GP) surgery, last data collection from the GP surgery, death or hospitalisation due to COVID-19.

### Case–control ascertainment

Cases were hospitalised for or died due to COVID-19 during follow-up, recorded as either a primary or secondary cause of hospitalisation or death. These outcomes were ascertained using the International Classification of Diseases (ICD)-10 codes (online supplemental table S1). We chose hospitalisation or death due to COVID-19 as a composite primary outcome as these are serious acute outcomes of COVID-19. We analysed them separately as well because mortality is a more severe outcome and, while the threshold for hospitalisation may vary over time and region, mortality is not susceptible to these variations.

The index date was the earliest date of hospitalisation or death due to COVID-19. For each case, up to ten controls matched by age (± 1 year), sex and number of days between cohort entry and index date, alive and contributing data to the CPRD on the date when a case experienced an outcome were selected by incidence-density (also called risk-set) sampling without replacement. In this sampling framework, the OR estimates the incidence rate ratio, which is practically equivalent to the HR in a piecewise constant hazard model.^22^ Matching on follow-up time provides an unbiased estimate of the HR that would be obtained from a time to event analysis of the full cohort, and its inherent time-dependent nature means that it is also free of immortal time bias.^23^ Matching each case to up to ten controls allowed the OR to be an appropriate estimate of the relative risk.^24^

### Exposures

The number of COVID-19 booster vaccinations (0–3) was the main exposure (online supplemental figure S1). Vaccinations against COVID-19 during each of the booster campaigns (ascertained using the start and end date of each campaign) and time since latest COVID-19 booster vaccination categorised as 0–13, 14–90, 91–180, 181–270, 271–365 and ≥366 days were secondary exposures. During the autumn 2021 and spring 2022 booster campaigns, the full-dose Pfizer BNT162b2 vaccine was predominantly used, with some individuals receiving half-dose Moderna mRNA1273.^14^ For the autumn 2022 booster campaign, the Pfizer/BioNTech bivalent Comirnaty Original/Omicron BA.1 booster and the Moderna Spikevax Original/Omicron BA.1 bivalent booster were used.^14^

### Negative control exposure

Eye or ear examination in primary care was the negative control exposure. There is no plausible reason for eye or ear examination to be associated with serious COVID-19 outcomes. This assessed confounding due to healthcare-seeking bias that can cause spurious associations (online supplemental table S2).^25^

### Covariates

Potential confounders included age (years); sex; body mass index (BMI, kg/m²); general practice-level Index of Multiple Deprivation (IMD) (quintile), an area-based measure of socioeconomic status derived from the postcode of the practice^26^; ethnicity (six-level ONS classification); smoking status (non-smoker, current, ex-smoker); alcohol consumption (non-alcohol, ex-alcohol use, low<14 units/week, moderate 14–21 units/week, excess>21 units/week); Charlson Comorbidity Index (0, 1, 2, 3, ≥4); other comorbidities that can cause severe COVID-19 (Addison’s disease, aplastic anaemia/haemoglobinopathy, atrial fibrillation, ischaemic heart disease, chronic lung disease, chronic mental illness, chronic neurological disease); oral corticosteroid prescription within ≤90 days of index date; SARS-CoV-2 infection and/or COVID-19 recorded either in primary care records or in hospital discharge summary as a clinical diagnosis and/or a positive result before start of follow-up; number of primary-care consultations in the year before start of follow-up (quintiles) and number of hospital admissions in the year before study start (quintiles).^26 27^

Since lifestyle factors such as smoking, BMI and alcohol intake are recorded opportunistically in primary care, we did not impose a fixed look-back period. The latest record was considered. We did not include the number of prior COVID-19 vaccines before start of follow-up as a covariate because approximately 97% of participants (cases and controls) had two primary vaccine doses.

### Statistical **analysis**

BMI, ethnicity, smoking and alcohol intake were the only covariates with missing data. These data were considered missing at random in a population with long-term inflammatory conditions and immune-suppressing drug prescription. Multiple imputation handled the missing data using chained equations. 20 imputations were undertaken using predictive mean matching (k = 20 neighbours) for BMI and multinomial logistic regression with augmentation for categorical variables, after 10 burn-in iterations^28^ . Imputation models included the case–control indicator, exposure and all covariates listed above using the command ‘mi impute’.

Descriptive statistics were presented as mean±SD for continuous variables or as n (%) for categorical variables. Standardised mean difference>0.10 was considered as covariate imbalance between cases and controls.^29^ Multivariable unconditional logistic regression estimated adjusted ORs and 95% CIs to assess the associations. Models were fitted using the ‘mi estimate: logistic’ command and results were combined using Rubin’s rule.

Three nested models were developed: model 1—age, sex and follow-up time; model 2—model 1 + BMI, IMD,^26^ alcohol, Charlson Comorbidity Index (CCI), ethnicity, smoking, oral corticosteroids and prior SARS-CoV-2 infection and/or COVID-19; and model 3: model 2 + primary care consultations and hospital admissions within 12 months of index date and at-risk conditions. 

The number of prior boosters received was included as an additional covariate in models 2 and 3 when evaluating the association between hospitalisation and/or death due to COVID-19 during the spring 2022, autumn 2022 booster campaigns.

Adjusted vaccine effectiveness (aVE) and 95% CI were calculated as (1 – adjusted OR (aOR)) × 100% using the aOR from model 3. For an association between hospitalisation or death due to COVID-19 and booster vaccination, the 95% CIs for aVE should not include 0%. Absolute risk difference was calculated in a single randomly selected dataset using model 3 covariates.

### Subgroup analysis

The association between primary outcome and the main exposure was stratified for sex, age (< 65, ≥65 years), SARS-CoV-2 infection or COVID-19 prior to the start of follow-up, IMIDs and immune-suppressing medications prescribed using model 3. Patients could be diagnosed with >1 IMID and prescribed >1 immune-suppressing drug. Each of them qualified the patients to be included in a relevant subgroup. We did not perform formal interaction tests and the subgroup analyses are exploratory and hypothesis generating rather than definitive.

### Sensitivity analysis

Although control for the matching factors can be obtained with no loss of validity and a possible increase in precision using an unconditional analysis provided there are no problems of sparse data^30^, we undertook conditional logistic regression as a sensitivity analysis using the fully adjusted model for hospitalisation and/or death due to COVID-19. Additional sensitivity analyses excluded patients with SARS-CoV-2 infection or COVID-19 prior to study start, outcomes in which there was a positive test for SARS-CoV-2 but no ICD-10 codes indicating COVID-19 and of patients who had received two COVID-19 vaccine doses prior to study start to represent a complete primary course. Data management and analyses were performed using Stata V.18 SE (StataCorp, College Station, Texas, USA).

### Patient and public involvement

This study was motivated by people with IMIDs. They recommended the use of routinely collected data instead of conducting a resource-intensive clinical trial.

## Results

**Figure 1:**
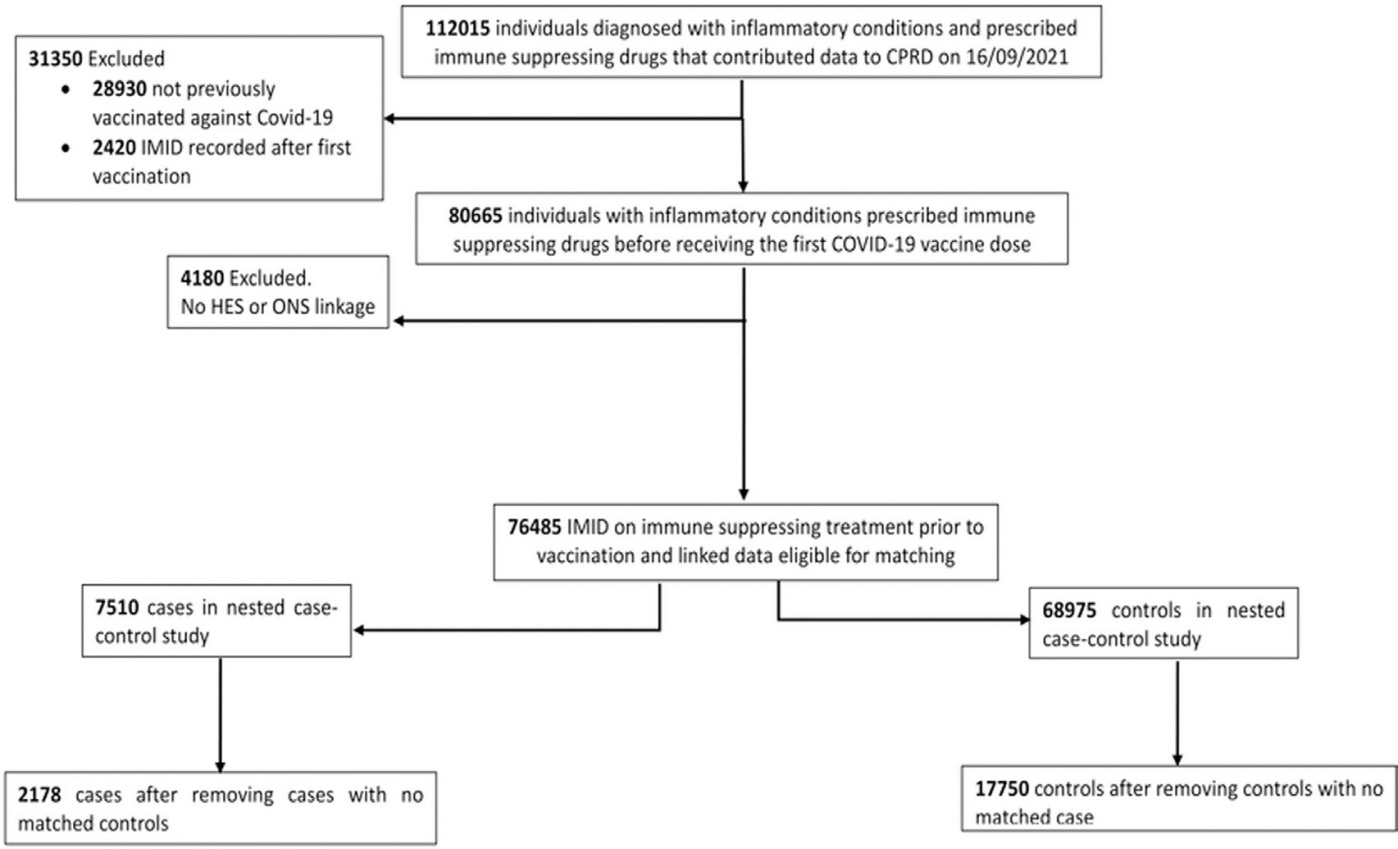
Patient selection flow chart. CPRD, Clinical Practice Research Datalink; HES, Hospital Episode Statistics; IMID, immune-mediated inflammatory diseases; ONS, Office for National Statistics.

19 928 people with IMIDs, including 2178 cases and 17 750 controls, were included (figure 1). Approximately 97% (96.4% of cases vs 97.1% of controls) had received two vaccine doses against COVID-19 prior to cohort entry. Cases had a slightly higher mean age than controls (66.6 (SD 16.4) vs 65.2 (SD 16.2) years). The distribution of sex, ethnicity and socioeconomic status was similar. Cases were more often current or former smokers (48.8% vs 45.8%) and were more likely to consume >21 units of alcohol per week (20.5% vs 16.5%) than controls. Cases had a greater comorbidity burden and a higher prevalence of chronic respiratory disease (8.0% vs 4.0%), ischaemic heart disease (16.8% vs 12.7%), atrial fibrillation (16.9% vs 10.9%) and chronic neurological disease (4.8% vs 2.8%) than controls. Cases were more frequently prescribed oral corticosteroids (27.5% vs 11.4%) than controls. Cases were more likely to use healthcare services, with a greater number of GP consultations (35.6 vs 25.6) and hospitalisations (3.1 vs 0.9) in the previous 12 months than controls. Prior COVID-19 or SARS-CoV-2 infection was less frequent among cases (3.2%) than among controls (6.0%) (table 1).

**Table 1:** Characteristics of cases and controls

	Controls (n=17 750)	Cases (n=2178)	SMD
Demographic details			
Age (years), mean±SD	65.2±16.2	66.6±16.4	0.09
BMI (kg/m^2^), mean±SD	27.8±6.1	28.2±6.9	0.06
Missing BMI	661 (3.7%)	69 (3.1%)	−0.03
Sex			
Male	7008 (39.5%)	873 (40.1%)	
Female	10 742 (60.5%)	1305 (59.9%)	−0.01
Follow-up time			
Time to index date (days); mean±SD	235.7 (135.0)	236.7 (135.1)	0.01
Ethnicity			
White	15 857 (89.3%)	1971 (90.5%)	0.04
Mixed	94 (0.5%)	12 (0.6%)	0.00
Bangladeshi/Indian/Pakistani	750 (4.2%)	97 (4.5%)	0.01
Black	244 (1.4%)	28 (1.3%)	−0.01
Chinese/other Asian	226 (1.3%)	27 (1.2%)	0.00
Other	553 (3.1%)	42 (1.9%)	−0.01
Missing	26 (0.1%)	-/- (0.0%)	−0.03
Index of Multiple Deprivation (quintiles)			
1 (least deprived)	3443 (19.4%)	369 (16.9%)	−0.06
2	3278 (18.5%)	415 (19.1%)	0.02
3	3820 (21.5%)	510 (23.4%)	0.05
4	3633 (20.5%)	463 (21.3%)	0.02
5 (most deprived)	3576 (20.1%)	421 (19.3%)	−0.02
Smoking status			
Non-smoker	9582 (54.0%)	1108 (51.0%)	−0.06
Current smoker	1659 (9.4%)	176 (7.9%)	−0.04
Ex-smoker	6457 (36.4%)	890 (40.9%)	0.09
Missing	52 (0.3%)	-/- (0.2%)	−0.02
Alcohol intake			
Non-drinker	3553 (20.0%)	539 (24.7%)	0.11
Ex-drinker	7828 (44.1%)	827 (38.0%)	−0.12
Low (<14 units/week)	877 (4.9%)	104 (4.8%)	−0.01
Moderate (14–21 units/week)	1072 (6.0%)	110 (5.1%)	−0.04
Excess (>21 units/week)	2932 (16.5%)	446 (20.5%)	0.10
Missing	1488 (8.4%)	152 (7.0%)	−0.05
Charlson Comorbidity Index			
0	3981 (22.4%)	284 (13.0%)	−0.25
1	5313 (29.9%)	442 (20.3%)	−0.22
2	2250 (12.7%)	331 (15.2%)	0.07
3	2357 (13.3%)	313 (14.4%)	0.03
≥4	3849 (21.7%)	808 (37.1%)	0.34
Clinical risk groups			
Chronic respiratory disease	702 (4.0%)	175 (8.0%)	0.17
Ischaemic heart disease	2250 (12.7%)	365 (16.8%)	0.12
Atrial fibrillation	1927 (10.9%)	368 (16.9%)	0.18
Chronic neurological disease	499 (2.8%)	105 (4.8%)	0.11
Addison’s disease	25 (0.1%)	9 (0.4%)	0.05
Asplenia/spleen dysfunction	170 (1.0%)	42 (1.9%)	0.08
Severe mental illness	124 (0.7%)	21 (1.0%)	0.03
Prior SARS-CoV-2 infection and/or COVID-19 (primary care or hospital) before start of follow-up			
No	16 679 (94.0%)	2108 (96.8%)	
Yes	1071 (6.0%)	70 (3.2%)	−0.13
Oral corticosteroid prescription*†			
No	15 721 (88.6%)	1580 (72.5%)	
Yes	2029 (11.4%)	598 (27.5%)	0.41
Healthcare utilisation			
GP consultations in last 12 months, mean±SD	25.6±13.8	35.6±18.0	0.62
Hospital admissions in last 12 months, mean±SD	0.9±2.4	3.1±5.2	0.54
Immune-suppressive drug prescription*†			
Methotrexate	7192 (40.5%)	736 (33.8%)	−0.14
5-amino salicylates/sulfasalazine	7339 (41.3%)	693 (31.8%)	−0.20
Thiopurine (azathioprine/6-mercaptopurine)	1772 (10.0%)	263 (12.1%)	0.07
Mycophenolate mofetil	281 (1.6%)	114 (5.2%)	0.20
Leflunomide	589 (3.3%)	67 (3.1%)	−0.01
Tacrolimus	46 (0.3%)	22 (1.0%)	0.09
Ciclosporin	41 (0.2%)	16 (0.7%)	0.07
None	2 199 (12.4%)	471 (21.6%)	0.25
Immune-mediated inflammatory diseases*			
Rheumatoid arthritis	8274 (46.6%)	1076 (49.4%)	0.06
Inflammatory bowel disease	6513 (36.7%)	654 (30.0%)	−0.14
Atopic dermatitis	3911 (22.0%)	525 (24.1%)	0.05
Psoriasis	2549 (14.4%)	338 (15.5%)	0.03
Psoriatic arthritis	1695 (9.6%)	184 (8.5%)	−0.03
Polymyalgia rheumatica	1071 (6.0%)	145 (6.7%)	0.03
Vasculitis	458 (2.6%)	106 (4.9%)	0.12
Systemic lupus erythematosus	353 (2.0%)	99 (4.6%)	0.14
Other connective-tissue disease	393 (2.2%)	97 (4.5%)	0.12
Giant cell arteritis	256 (1.4%)	44 (2.0%)	0.04
Axial spondyloarthritis	260 (1.5%)	32 (1.5%)	0.00
Reactive arthritis	107 (0.6%)	27 (1.2%)	0.07

* The total was greater than the number of cases (controls) as some patient could be prescribed multiple immune-suppressing drugs or be diagnosed with multiple immune-mediated inflammatory diseases.

† Within 90 days of the index date.

-/-, Number <5 suppressed as per CPRD instructions to prevent patient re-indetification; BMI, body mass index; GP, General Practice; IBD, inflammatory bowel disease; RA, rheumatoid arthritis; SLE, systemic lupus erythematosus; SMD, standardised mean difference.

Hospitalisation and/or death due to COVID-19 was negatively associated with three (aOR (95% CI) 0.20 (0.15 to 0.28)), two (aOR (95% CI) 0.29 (0.23 to 0.36)) or one booster dose (aOR (95% CI) 0.56 (0.48 to 0.65)) respectively, compared with no booster doses (table 2). The corresponding adjusted risk difference (RD) (95% CI) was −0.06 (−0.07 to −0.04), −0.10 (−0.12 to −0.08) and −0.12 (−0.14 to −0.10), respectively. A similar pattern was observed when hospitalisation and death were considered separately, although the CIs were wide for death due to the small number of events (table 2). Hospitalisation and/or death due to COVID-19 was not associated with eye or ear examination (aOR (95% CI) 0.98 (0.81 to 1.18)) (table 2).

**Table 2:** Hospitalisation and/or death due to COVID-19 and number of COVID-19 booster vaccinations

Outcome	Cases	Controls	Model 1 aOR (95% CI)*	Model 2 aOR (95% CI)†	Model 3 aOR (95% CI)‡	aVE (95% CI)%‡	aRD (95% CI)‡
Hospitalisation or death due to COVID-19	n=2178	n=17 750					
0 dose	379	2093	1	1	1		
1 dose	1344	10 894	0.55 (0.48 to 0.62)	0.55 (0.48 to 0.63)	0.56 (0.48 to 0.65)	44 (35 to 52)	−0.06 (−0.07 to −0.04)
2 doses	325	3247	0.30 (0.25 to 0.37)	0.29 (0.24 to 0.36)	0.29 (0.23 to 0.36)	71 (64 to 77)	−0.10 (−0.12 to −0.08)
3 doses	130	1516	0.20 (0.15 to 0.26)	0.20 (0.15 to 0.26)	0.20 (0.15 to 0.28)	80 (72 to 85)	−0.12 (−0.14 to −0.10)
Hospitalisation due to COVID-19§¶	n=1986	n=17 750					
0 dose	316	2093	1	1	1		
1 dose	1246	10 894	0.61 (0.53 to 0.71)	0.61 (0.52 to 0.70)	0.61 (0.53 to 0.72)	39 (28 to 47)	−0.04 (−0.06 to −0.03)
2 doses	308	3247	0.35 (0.29 to 0.43)	0.34 (0.27 to 0.42)	0.33 (0.26 to 0.42)	67 (58 to 74)	−0.09 (−0.11 to −0.09)
3 doses	116	1516	0.22 (0.17 to 0.30)	0.22 (0.16 to 0.29)	0.22 (0.16 to 0.30)	78 (70 to 84)	−0.11 (−0.13 to −0.09)
Death due to COVID-19§	n=192	n=17 750					
0 dose	63	2093	1	1	1		
1 dose	98	10 894	0.22 (0.15 to 0.31)	0.24 (0.17 to 0.35)	0.23 (0.15 to 0.34)	77 (66 to 85)	−0.03 (−0.04 to −0.02)
2 doses	17	3247	0.07 (0.04 to 0.13)	0.08 (0.04 to 0.15)	0.07 (0.03 to 0.14)	93 (86 to 97)	−0.04 (−0.05 to −0.02)
3 doses	14	1516	0.08 (0.04 to 0.18)	0.11 (0.05 to 0.24)	0.10 (0.04 to 0.23)	90 (77 to 96)	−0.04 (−0.05 to −0.02)
Negative control exposure eye or ear examination	n=2178	n=17 750					
No	2009	16 544	1	1	1		
Yes	169	1206	1.13 (0.96 to 1.33)	1.05 (0.89 to 1.25)	0.98 (0.81 to 1.18)	–/–	–/–

* Adjusted for age, sex and duration of follow-up.

† Adjusted for age, sex, duration of follow-up, body mass index (BMI), Charlson’s Comorbidity Index, ethnicity, deprivation (Index of Multiple Deprivation quintile), alcohol intake, smoking status, oral corticosteroid prescription in the 90 days prior to the index date and prior SARS-CoV-2 infection and/or COVID-19 before start of follow-up.

‡ Adjusted as in model 2 plus healthcare utilisation (general practice consultations and hospital admissions in the previous 12 months, quintiles) and clinical risk group status. Adjusted vaccine effectiveness (aVE (95% CI)) was calculated as 1−aOR (95% CI) from model 3. Adjusted risk difference (aRD) was obtained from model 3 using data from a single randomly selected imputed dataset.

§ A small number died on the date of hospital admission.

¶ Cases who died due to COVID-19 before hospitalisation were excluded.

aOR, adjusted OR.

Receipt of a booster in autumn 2021, spring 2022 or autumn 2022 individually was associated with reduced odds of hospitalisation and/or death due to COVID-19, with similar aOR and aRD across the three cycles (table 3). Hospitalisation and/or death due to COVID-19 was negatively associated with booster vaccination received within 365 days. The aOR (95% CI) for hospitalisation and/or death due to COVID-19 and booster vaccination between 1–13, 14–90, 91–180, 181–270 and 271–365 days were 0.35 (0.26 to 0.48), 0.52 (0.44 to 0.61), 0.64 (0.54 to 0.76), 0.76 (0.60 to 0.95) and 0.62 (0.45 to 0.86), respectively, compared with no booster vaccination. The corresponding aRD (95% CI) were −0.08 (−0.10 to −0.06), −0.06 (−0.07 to −0.04), −0.04 (−0.06 to −0.02), −0.03 (−0.05 to −0.00) and −0.04 (−0.07 to −0.02), respectively. There was no statistically significant association after 365 days (aOR (95% CI) 1.22 (0.82 to 1.83) and aRD (95% CI) 0.02 (−0.02 to 0.06)) (table 4). Analyses also showed broadly similar pattern for hospitalisation and death separately.

**Table 3:** Hospitalisation and/or death due to COVID-19 and vaccination during individual booster campaigns

Outcome	Booster campaign window (dates)	Booster	Cases	Controls	Model 1 aOR (95% CI)*	Model 2 aOR (95% CI)†	Model 3 aOR (95% CI)‡	aVE (95% CI)%‡	aRD (95% CI)‡
Hospitalisation or death due to COVID-19	Autumn 2021 (20 September 2021 to 20 March 2022)	No	310	1726	1	1	1		
		Yes	630	6075	0.40 (0.33 to 0.48)	0.39 (0.32 to 0.47)	0.40 (0.32 to 0.50)	60 (50 to 68)	−0.08 (−0.10 to −0.06)
	Spring 2022 (21 March 2022 to 04 September 2022)	No	626	444	1	1	1		
		Yes	163	1924	0.51 (0.42 to 0.62)	0.51 (0.41 to 0.62)	0.52 (0.42 to 0.65)	48 (35 to 58)	−0.05 (−0.06 to −0.03)
	Autumn 2022 (05 September 2022 to 04 March 2023)	No	252	1207	1	1	1		
		Yes	167	2135	0.30 (0.24 to 0.38)	0.31 (0.24 to 0.39)	0.33 (0.25 to 0.43)	67 (57 to 75)	−0.09 (−0.11 to −0.07)
Hospitalisation due to COVID-19§	Autumn 2021 (20 September 2021 to 20 March 2022)	No	25	1726	1	1	1		
		Yes	585	6075	0.44 (0.36 to 0.53)	0.42 (0.34 to 0.52)	0.43 (0.34 to 0.53)	57 (47 to 66)	−0.07 (−0.09 to −0.05)
	Spring 2022 (21 March 2022 to 04 September 2022)	No	584	4440	1	1	1		
		Yes	156	1924	0.53 (0.43 to 0.65)	0.53 (0.43 to 0.65)	0.54 (0.44 to 0.68)	46 (32 to 56)	−0.04 (−0.06 to −0.03)
	Autumn 2022 (05 September 2022 to 04 March 2023)	No	230	1207	1	1	1		
		Yes	153	2135	0.31 (0.25 to 0.39)	0.32 (0.25 to 0.40)	0.33 (0.25 to 0.44)	67 (56 to 75)	−0.08 (−0.10 to −0.06)
Death due to COVID-19¶	Autumn 2021 (20 September 2021 to 20 March 2022)	No	56	1726	1	1	1		
		Yes	45	6075	0.16 (0.09 to 0.29)	0.17 (0.09 to 0.30)	0.17 (0.09 to 0.33)	83 (67 to 91)	−0.03 (−0.05 to −0.02)
	Spring 2022 (21 March 2022 to 04 September 2022)	No	42	4440	1	1	1		
		Yes	7	1924	0.22 (0.09 to 0.50)	0.23 (0.10 to 0.54)	0.22 (0.10 to 0.54)	78 (46 to 90)	−0.01 (−0.01 to −0.00)
	Autumn 2022 (05 September 2022 to 04 March 2023)	No	22	1207	1	1	1		
		Yes	14	2135	0.25 (0.12 to 0.50)	0.29 (0.14 to 0.61)	0.31 (0.14 to 0.68)	69 (32 to 86)	−0.01 (−0.02 to −0.00)

* Adjusted for age, sex and duration of follow-up.

† Adjusted for age, sex, duration of follow-up, body mass index, Charlson’s Comorbidity Index, ethnicity, deprivation (Index of Multiple Deprivation quintile), alcohol intake, smoking status, oral corticosteroid prescription in the 90 days prior to the index date, prior SARS-CoV-2 infection and/or COVID-19 before start of follow-up and prior booster.

‡ Adjusted as in model 2 plus healthcare utilisation (general practice consultations and hospital admissions in the previous 12 months, quintiles), clinical risk group status and prior boosters. Adjusted vaccine effectiveness (aVE) (95% CI) was calculated as 1−aOR (95% CI) from model 3. Adjusted risk difference (aRD) was obtained from model 3 using data from a single randomly selected imputed dataset.

§ A small number died on the date of hospital admission.

¶ Cases who died due to COVID-19 before hospitalisation were excluded.

aOR, adjusted OR.

**Table 4:** Hospitalisation and/or death due to COVID-19 and time since most recent booster vaccination

Outcome	Time since latest booster	Cases	Controls	Model 1 aOR (95% CI)*	Model 2 aOR (95% CI)†	Model 3 aOR (95% CI)‡	aVE (95% CI)%‡	aRD (95% CI)‡
Hospitalisation or death due to COVID-19	No booster	379	2093	1	1	1		
	0–13 days	61	1011	0.32 (0.24 to 0.42)	0.34 (0.25 to 0.45)	0.35 (0.26 to 0.48)	65 (52 to 74)	−0.08 (−0.10 to −0.06)
	14–90 days	613	6541	0.50 (0.43 to 0.58)	0.50 (0.43 to 0.58)	0.52 (0.44 to 0.61)	48 (39 to 56)	−0.06 (−0.07 to −0.04)
	91–180 days	736	5685	0.66 (0.57 to 0.76)	0.63 (0.54 to 0.74)	0.64 (0.54 to 0.76)	36 (24 to 46)	−0.04 (−0.06 to −0.02)
	181–270 days	242	1457	0.90 (0.74 to 1.09)	0.80 (0.65 to 0.99)	0.76 (0.60 to 0.95)	24 (5 to 40)	−0.03 (−0.05 to −0.00)
	271–365 days	80	560	0.78 (0.59 to 1.03)	0.66 (0.48 to 0.89)	0.62 (0.45 to 0.86)	38 (14 to 55)	−0.04 (−0.07 to −0.02)
	≥366 days	67	223	1.63 (1.19 to 2.24)	1.21 (0.85 to 1.72)	1.22 (0.82 to 1.83)	−22 (−83 to 18)	0.02 (−0.02 to 0.06)
Hospitalisation due to COVID-19§¶	No booster	316	2093	1	1	1		
	0–13 days	58	1011	0.37 (0.27 to 0.49)	0.39 (0.29 to 0.52)	0.40 (0.29 to 0.54)	60 (46 to 71)	−0.07 (−0.09 to −0.05)
	14–90 days	577	6541	0.56 (0.48 to 0.66)	0.56 (0.48 to 0.65)	0.58 (0.49 to 0.68)	42 (32 to 51)	−0.04 (−0.06 to −0.03)
	91–180 days	676	5685	0.73 (0.62 to 0.85)	0.69 (0.59 to 0.81)	0.69 (0.58 to 0.83)	31 (17 to 42)	−0.03 (−0.05 to −0.02)
	181–270 days	228	1457	0.99 (0.82 to 1.21)	0.86 (0.70 to 1.07)	0.81 (0.64 to 1.03)	19 (−3 to 36)	−0.02 (−0.04 to −0.00)
	271–365 days	76	560	0.86 (0.64 to 1.14)	0.70 (0.51 to 0.95)	0.66 (0.47 to 0.92)	34 (8 to 53)	−0.03 (−0.06 to −0.01)
	≥366 days	55	223	1.53 (1.09 to 2.16)	1.07 (0.73 to 1.57)	1.03 (0.68 to 1.58)	-3 (−58 to 32)	0.00 (−0.04 to 0.04)
Death due to COVID-19^¶^	No booster	63	2093	1	1	1		
	0–13 days	‡	1011	0.07 (0.02 to 0.24)	0.09 (0.03 to 0.30)	0.10 (0.03 to 0.33)	90 (67 to 93)	−0.02 (−0.03 to −0.02)
	14–90 days	36	6541	0.16 (0.10 to 0.26)	0.19 (0.12 to 0.29)	0.19 (0.12 to 0.31)	81 (69 to 88)	−0.02 (−0.03 to −0.01)
	91–180 days	60	5685	0.28 (0.18 to 0.44)	0.32 (0.21 to 0.50)	0.32 (0.20 to 0.51)	68 (49 to 80)	−0.02 (−0.03 to −0.01)
	181–270 days	14	1457	0.37 (0.19 to 0.70)	0.39 (0.20 to 0.77)	0.36 (0.18 to 0.73)	64 (27 to 82)	−0.02 (−0.03 to −0.01)
	271–365 days	-/-	560	0.36 (0.12 to 1.04)	0.38 (0.13 to 1.13)	0.31 (0.10 to 0.97)	69 (3 to 90)	−0.02 (−0.03 to −0.00)
	≥366 days	12	223	2.22 (1.07 to 4.63)	2.09 (0.95 to 4.60)	2.01 (0.83 to 4.89)	−101 (−389 to 17)	0.02 (−0.01 to 0.05)

* Adjusted for age, sex and duration of follow-up.

† Adjusted for age, sex, duration of follow-up, body mass index, Charlson’s Comorbidity Index, ethnicity, deprivation (Index of Multiple Deprivation quintile), alcohol intake, smoking status, oral corticosteroid prescription in the 90 days prior to the index date, prior SARS-CoV-2 infection and/or COVID-19 before start of follow-up and prior boosters.

‡ Adjusted as in model 2 plus healthcare utilisation (general practice consultations and hospital admissions in the previous 12 months, quintiles), clinical risk group status and prior boosters. Adjusted vaccine effectiveness (aVE) (95% CI) was calculated as 1−aOR (95% CI) from model 3. Adjusted risk difference (aRD) was obtained from model 3 using data from a single randomly selected imputed dataset.

§ A small number died on the date of hospital admission.

¶ .Cases who died due to COVID-19 before hospitalisation were excluded.

** -/- Number <5 suppressed as per CPRD instructions to prevent patient re-indetification.

aOR, adjusted OR.

### Subgroup analysis

**Figure 2:**
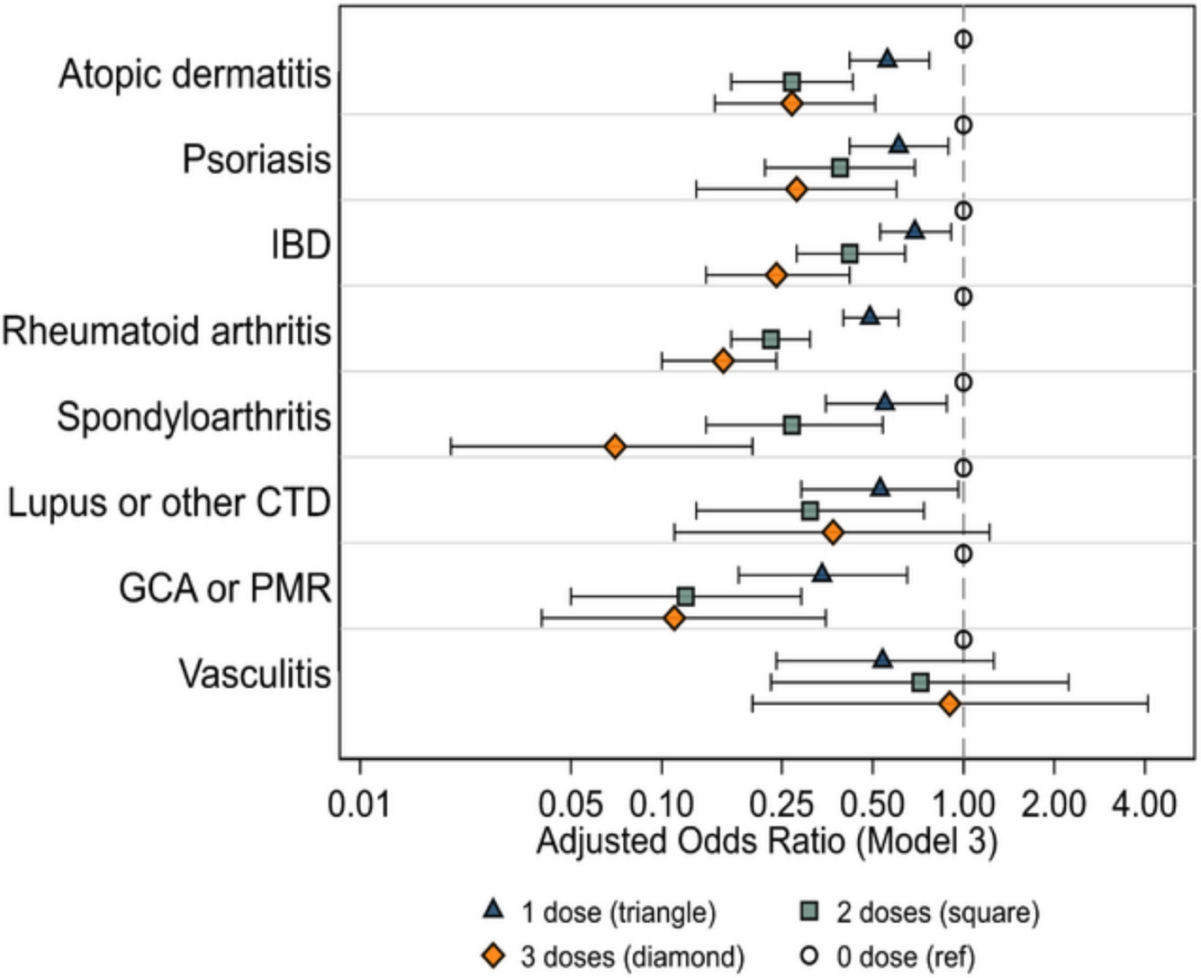
Adjusted ORs (model 3) for the association between the number of COVID-19 booster vaccinations and COVID-19 hospitalisation and/or death, stratified by immune-mediated inflammatory diseases. Booster vaccinations were administered after the completion of primary vaccination roll-out in the UK. CTD, connective tissue disease; GCA, Giant cell arteritis; IBD, inflammatory bowel disease; PMR, Polymyalgia rheumatica.

Results were consistent across most IMID categories, although some heterogeneity was observed (figure 2). There was a negative association between hospitalisation and/or death due to COVID-19 and the number of boosters against COVID-19 among patients with RA; giant cell arteritis or polymyalgia rheumatica; AxSpA; atopic dermatitis; or psoriasis (online supplemental table S3). There was no such association in SLE, other connective tissue diseases or vasculitis, potentially due to the small sample size.

**Figure 3:**
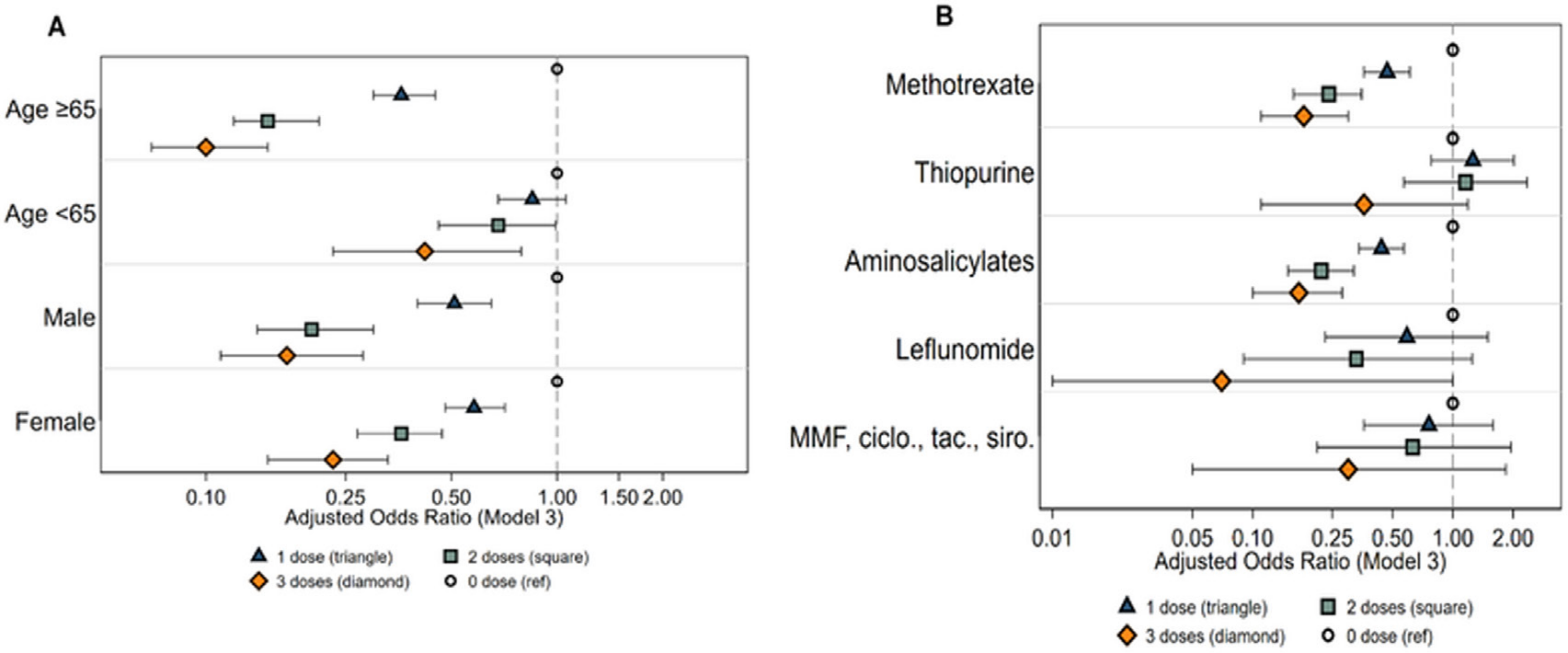
Adjusted ORs (model 3) for the association between the number of COVID-19 booster vaccinations and COVID-19 hospitalisation and/or death, stratified by (A) age and sex and by (B) immunosuppressive drug. Booster vaccinations were administered after the completion of primary vaccination roll-out in the UK. Ciclo, Ciclosporin; MMF, Mycophenolate mofetil; Siro, Sirolimus; tac, Tacrolimus

Among people aged at least 65 years, there was a negative association between hospitalisation and/or death due to COVID-19 and one, two or three booster vaccinations against COVID-19 (figure 3A and online supplemental table S3). Conversely, for individuals younger than 65 years, the negative association was evident only for two or three boosters. The associations were consistent across both sexes. When the analyses were stratified by immune-suppressive drugs prescribed within 90 days of the index date, the negative associations were observed for methotrexate and sulfasalazine/5-aminosalicylate but not for leflunomide and other potent immune-suppressing drugs . Individuals prescribed thiopurines had no significant association (figure 3B and online supplemental table S4).

Conditional logistic regression models gave very similar estimates for the associations between hospitalisation and/or death due to COVID-19 and number of boosters, individual boosters and time since latest booster (online supplemental table S5). Results were unchanged on excluding those recorded to have previous SARS-CoV-2 infection or COVID-19, excluding those who had received one primary COVID-19 vaccine dose and when the outcome was restricted to COVID-19 recorded as a primary discharge diagnosis (online supplemental tables S6–S8).

### Discussion

This study demonstrated that hospitalisation and/or death due to COVID-19 were negatively associated with an increasing number of booster vaccinations and with individual booster vaccinations in adults with IMIDs. The magnitude of the association was comparable across both sexes and the association was present in those older than 65 years of age. The odds of death due to COVID-19 were substantially lower than the odds of hospitalisation due to COVID-19. However, this should be interpreted with caution due to potential for residual confounding and the healthy vaccinee effect. While patients with rheumatic and skin diseases demonstrated a negative association between hospitalisation and death due to COVID-19 and booster doses, for those with multisystem illnesses such as vasculitis or SLE there was no such association. This could be driven by the small number of patients with vasculitis or SLE and by the fact that they are more likely to be prescribed anti-B-cell therapies that inhibit vaccine-induced immunity.

The autumn 2022 campaign primarily used bivalent mRNA boosters^31^ and the larger magnitude of risk reduction in this vaccination campaign was expected. The aVE of booster vaccinations in this study was more than that reported in general population studies from England in 2022/2023.^32^ This could have occurred as our study population was clinically vulnerable and experienced a greater relative risk reduction. Nevertheless, the aVE in our study was similar to that reported in a study from Nordic countries that included data from people with a broad range of IMIDs.^33^

To our knowledge, this is the first study to evaluate the association between serious COVID-19 outcomes and COVID-19 booster vaccinations in a range of IMIDs using nationwide data from England, and to evaluate the association with an increasing number of booster vaccinations. The key strengths of our study stem from the use of CPRD, a nationally representative dataset of routine primary care records in the National Health Service (NHS). Healthcare in the NHS is free at the point of use for all UK residents. We ascertained people with a range of IMIDs. These factors increase the generalisability of our findings. CPRD has been extensively used for medical research and has a high degree of validity for diagnoses of long-term conditions including autoimmune conditions.^34^ Vaccinations are undertaken in primary care in the UK and are recorded in the CPRD. Cases were defined using ICD-10 codes recorded in hospitalisation and mortality records. In the UK, hospital discharge summaries and death certification are completed by doctors and have high face validity. Additionally, the analysis adjusted for a range of confounders and used multiple imputation methods to account for missing data, with similar results across multiple sensitivity analyses. The lack of association between hospitalisation and/or death due to COVID-19 and eye or ear examination suggests a lack of healthcare-seeking bias and residual confounding. This supports the validity of our findings.

Nevertheless, our study has limitations. First, since our study used primary care data to identify boosters, people vaccinated in other settings were considered unexposed, potentially biasing the association towards null. We do not believe this is a major issue because COVID-19 boosters could only be accessed through the NHS’s national vaccination programme during the study period. Second, since targeted synthetic drugs and biologics are prescribed only in hospitals, CPRD does not contain these prescription records, and we could not account for their use. Lack of adjustment for targeted synthetic drugs and biologics could overestimate risk reduction as they are more likely to seek vaccination and experience hospitalisation and death due to COVID-19. We could not report ORs for patients prescribed biologics or whose IMIDs are managed exclusively in hospitals. Third, our study did not include rare IMIDs such as systemic sclerosis or polymyositis, and further research is needed to examine whether our findings could apply to these patient groups. Fourth, while we adjusted analyses for several confounding variables, unmeasured confounding may have influenced the associations. Fifth, we were unable to conduct subgroup analyses for certain characteristics such as ethnicity due to a lack of sample size, and it is likely that estimates for certain rarer IMIDs such as vasculitis are imprecise due to few patients. Similarly, estimates for vaccine waning are also limited by sample size, as each category is mutually exclusive leading to reduced sample size in some strata. Sixth, we were unable to provide specific estimates for COVID-19 variants or the subsequent monovalent/bivalent boosters. Seventh, we were unable to comment on the association between vaccination and disease activity as disease-activity scores are not included in the CPRD. Eighth, due to lack of data on hospitalisation and mortality records beyond March 2023, we were unable to examine the effectiveness of more recent booster vaccinations. Ninth, as we defined the primary outcome as hospitalisation or death due to COVID-19, we did not specify whether this was a primary or secondary cause of hospitalisation or death and did not have any rule to exclude hospital-acquired infection, which may bias our findings. Tenth, we categorised time since last booster vaccination using intuitive intervals. While this makes the results easy to interpret, an alternate strategy would have been to treat this as a continuous variable using splines. Lastly, since we did not adjust for SARS-CoV-2 infections during the follow-up period, our estimates should be interpreted in the context of this potential unmeasured time-varying confounding. It is also important to acknowledge that due to the extensive use of self-testing, many SARS-CoV-2 infections will not be recorded in primary care data. It is possible that healthy vaccinee bias contributed to the large magnitude of negative association between hospitalisation or death due to COVID-19 and booster vaccinations. A healthy vaccinee bias occurs when someone who is otherwise well and has a healthy lifestyle seeks vaccination, potentially inflating vaccine effectiveness estimates in observational studies. We believe this bias is less likely to play a major role in an IMID population already prescribed steroid-sparing immune-suppressing drugs. This is supported by the presence of an expected waning of vaccine effectiveness over time. Nevertheless, our findings should be interpreted with caution considering this potential strong bias.

Our results corroborate emerging real-world evidence demonstrating booster effectiveness in immunosuppressed populations. Studies from the USA reported 37–51% relative reduced risk of COVID-19 related hospitalisation in systemic autoimmune rheumatic diseases during the Omicron wave.^35 36^ Similar results have been reported in a study using data from Mass General Brigham and from a registry in Israel.^37 38^ Our results build on these findings by providing evidence for a dose–response relationship, reporting analyses for individual at-risk groups and evaluating separate booster campaigns.

Immunogenicity studies in immunocompromised populations provide a mechanistic underpinning for our findings. The receipt of four or five COVID-19 vaccine doses was associated with antibody positivity in participants with solid organ transplants, rare IMIDs and lymphoid malignancies compared with receiving only three vaccine doses.^18^ Importantly, boosters do more than restore circulating antibody: Furer *et al* reported renewed germinal-centre activity, expansion of class-switched memory B cells and increased spike-specific CD4^+^/CD8^+^ T-cell frequencies after the third dose in rheumatic-disease patients, providing biological plausibility for the incremental risk reduction we detected.^39^ Immune-suppressing drugs such as methotrexate blunt vaccine-induced immunity.^40 41^ We observed effectiveness of the boosters in this population, providing clinically relevant data for guiding vaccination policy. Conversely, the lack of association in those prescribed thiopurines is surprising and merits further assessment. The non-significant and imprecise estimates in patients with SLE and vasculitis reflect the small sample sizes in our dataset and yet are biologically plausible given evidence of diminished serological responses in these diseases when high-dose glucocorticoids or B-cell-depleting agents are used.^42 43^ Patients without a primary-care immune-suppressing drug prescription within 90 days of the index date, who are likely to include those prescribed biologics, exhibited the same graded protection, suggesting that boosters can overcome the additional immunosuppressive burden of parenteral biologics after sufficient antigenic stimulation.^41^

Booster vaccine uptake is declining in IMID patients globally with concerns about safety and effectiveness being the key common addressable reasons.^17 44 45^ In our previous studies, we did not find any evidence that vaccination against COVID-19 was associated with flares of autoimmune rheumatic diseases, skin disease or IBD that required NHS help.^46^,^48^ Collectively, the gaps in patient confidence can be addressed using results from this and previous studies. They would need to be combined with clear clinician guidance and tailored patient education and support.

Our results support current UK recommendations for booster vaccinations, whereby the JCVI currently advises 6 monthly autumn and spring boosters for immunocompromised individuals. Similar recommendations have also been made in the USA.^49^ Our time-since-booster analysis demonstrated clear waning of protection over time, with the strongest association observed within the first 3 months and more modest protection up to 1 year. These findings align with serological evidence of declining neutralising activity after 6 months of booster receipt and highlight the importance of regular booster scheduling to maintain protection in immunocompromised patients.^50 51^ The lack of statistically significant association between hospitalisation and/or death due to COVID-19 and booster vaccination more than 365 days ago does not imply absence of any protection.

Further research is needed to characterise the booster-induced protection against emergent Omicron subvariants, vaccines developed in response (BA.4/BA.5, XBB) and in patients prescribed biologics and targeted synthetic drugs.

In conclusion, in people with IMIDs, hospitalisation and/or death due to COVID-19 was negatively associated with booster vaccinations against COVID-19. These findings underscore the importance of booster vaccination for people with IMIDs, and the results could be used to promote COVID-19 vaccination in this at-risk group.

## Supplementary material

10.1136/rmdopen-2026-006834 online supplemental file 1

## Data Availability

Data may be obtained from a third party and are not publicly available.
