# Supplementary material for: Association between SARS-CoV-2 booster vaccination and hospitalisation and/or death due to COVID-19 in adults with immune-mediated inflammatory diseases: nested case–control study using linked primary-care, hospitalisation and mortality data from England
Source: RMD Open. 2026 Jul 3;12(3):e006834. doi: 10.1136/rmdopen-2026-006834 (PMC13343080; doi:10.1136/rmdopen-2026-006834)
Supplement: online supplemental file 1 [file rmdopen-12-3-s001.docx]

**Supplementary material**

Table of Contents

Table S1: ICD-10 codes for hospitalisation or death due to COVID-19 2

Table S2: Medical codes for ascertaining negative control exposure 3

Table S3: Hospitalisation and/or death due to COVID-19 and number of COVID-19 booster vaccinations in different immune mediated inflammatory diseases 5

Table S4: Hospitalisation and/or death due to COVID-19 and number of COVID-19 booster vaccinations: subgroup analysis 6-7

Table S5: Conditional logistic regression model examining association between hospitalisation and/or death due to COVID-19 and COVID-19 booster vaccination 8

Table S6: Hospitalisation and/or death due to COVID-19 and number of COVID-19 booster vaccinations after excluding those with a primary-care or secondary-care record of SARSCoV-2 infection or COVID-19 prior to study start  9

Table S7: Hospitalisation and/or death due to COVID-19 and number of COVID-19 booster vaccinations excluding those with a positive test result alone  10

Table S8: Hospitalisation and/or death due to COVID-19 and number of COVID-19 booster vaccinations in patients who received a two-dose primary course of COVID-19 vaccination11

Figure S1: COVID-19 booster windows during study period 12

**Table S1: ICD-10 codes for hospitalisation or death due to COVID-19**

| icd10 | Description |
| --- | --- |
| Z51.5 | COVID-19 in palliative care |
| B34.2 | Coronavirus infection, unspecified site |
| B97.2 | Coronavirus as the cause of diseases classified to other chapters |
| B97.29 | Other coronavirus as the cause of diseases classified elsewhere |
| J12.81 | Pneumonia due to SARS-associated coronavirus |
| J12.82 | Pneumonia due to coronavirus disease 2019 |
| O98.5 | COVID-19 in pregnancy |
| U04.9 | Severe acute respiratory syndrome [SARS], unspecified |
| U07.1 | Covid virus identified |
| U07.3 | Multisystem inflammatory syndrome associated with COVID-19 |
| U07.5 | Multisystem inflammatory syndrome associated with COVID-19 |
| U10 | Multisystem inflammatory syndrome associated with COVID-19, unspecified |
| U49 | emergency code for emergent diseases |

**Table S2:** **Medical codes for ascertaining negative control exposure**

| Medcodeid | Description |
| --- | --- |
| **Eye examination** |  |
| 253768010 | O/E - general eye examination |
| 253792019 | O/E - general eye examn. NOS |
| 509334014 | Examination of eye under anaesthetic |
| 1160901000000114 | On examination - visual acuity right eye=3/4.5 |
| 1821741000006112 | On examination - left eye visual acuity - corrected |
| 1821751000006114 | On examination - right eye visual acuity - corrected |
| 3083911000006111 | Eye examination |
| 3083921000006115 | Examining eye |
| 3083941000006110 | Examination of eye |
| 3128221000006115 | Electromyogram examination of eye |
| 3981591000006117 | Eye examination under anaesthesia |
| 3981611000006111 | EUA - Examination of eye under anesthetic |
| 3981621000006115 | Examination of eye under anesthetic |
| 3981641000006110 | EUA - Examination of eye under anaesthetic |
| 4552681000006113 | On examination - general eye examination |
| 4572531000006111 | On examination - visual acuity left eye=6/18 |
| 4572651000006115 | On examination - left eye counts fingers only |
| 4574041000006116 | On examination - eye movements |
| 4574061000006117 | On examination - eye movements normal |
| 5509301000006117 | On examination - right eye perceives light only |
| 5509331000006113 | On examination - left eye perceives light only |
| 5531211000006118 | Eye examination interpretation |
| 5974821000006118 | On examination - right eye sees hand movements |
| 5974851000006110 | On examination - left eye sees hand movements |
| 5974861000006112 | On examination - L-eye sees hand movements |
| 6866521000006112 | On examination - left eye no maculopathy |
| 6866881000006114 | On examination - right eye no maculopathy |
| 6917511000006114 | On examination - pinhole visual acuity right eye |
| 6919391000006117 | On examination - cataract of left eye |
| 6923281000006117 | On examination - cataract of right eye |
| 8035441000006110 | Problem focused eye examination normal |
| 8047501000006115 | Eye and vision examination normal |
| **Ear examination** |  |
| 255572010 | Ear examination - normal |
| 267450010 | Examination of ear under anaesthetic |
| 461199010 | [V]Examination of ears and hearing |
| 1786152012 | Individual hearing examination |
| 916511000006114 | Hearing examination |
| 916521000006118 | Individual hearing examination |
| 1598651000000111 | Ear examination abnormal |
| 2348671000000113 | Hearing examination - no abnormality detected |
| 4650731000006113 | EUA - Examination of ear under anesthetic |
| 4650741000006115 | Examination of ear under anesthetic |
| 4650751000006118 | EUA - Examination of ear under anaesthetic |
| 4944861000006117 | Examination of ear |
| 4944871000006112 | Clinical examination of ear |
| 5033301000006114 | Examination of ear under microscope |
| 5033311000006112 | EUM ear - Examination of ear under microscope |
| 5532641000006113 | On examination - ear |
| 6598981000006118 | Hearing examination |
| 12457601000006118 | [V]Examination of ears and hearing |
| 14622881000006115 | Ear examination normal |

**Table S3 Hospitalisation and/or death due to COVID-19 and number of COVID-19 booster vaccinations in different immune mediated inflammatory diseases (IMIDs)**

| **IMID** | **Booster doses** | **Cases**  **n = 2, 178** | **Controls**  **n= 17, 750** | **Adjusted OR  (95 % CI)^1^** | **aVE (95 % CI)^1^** |
| --- | --- | --- | --- | --- | --- |
| Rheumatoid arthritis | 0 | 198 | 910 | 1 |  |
|  | 1 | 642 | 4,857 | 0.49 (0.40 – 0.61) | 51 (39 to 60) % |
|  | 2 | 169 | 1,692 | 0.23 (0.17 – 0.31) | 77 (69 to 83) % |
|  | 3 | 67 | 815 | 0.16 (0.10 – 0.24) | 84 (76 to 90) % |
| Giant cell arteritis or polymyalgia rheumatica | 0 | 26 | 90 | 1 |  |
|  | 1 | 93 | 649 | 0.34 (0.18 – 0.65) | 66 (35 to 82) % |
|  | 2 | 31 | 304 | 0.12 (0.05 – 0.29) | 88 (71 to 95) % |
|  | 3 | 19 | 172 | 0.11 (0.04 – 0.35) | 89 (65 to 96) % |
| Lupus or other connective tissue diseases | 0 | 27 | 70 | 1 |  |
|  | 1 | 122 | 458 | 0.53 (0.29 – 0.96) | 47 (4 to 71) % |
|  | 2 | 30 | 150 | 0.31 (0.13 – 0.74) | 69 (26 to 87) % |
|  | 3 | 11 | 53 | 0.37 (0.11 – 1.22) | 63 (-22 to 89) % |
| Axial Spondyloarthritis | 0 | 39 | 242 | 1 |  |
|  | 1 | 153 | 1,266 | 0.55 (0.35 – 0.88) | 45 (12 to 65) % |
|  | 2 | 37 | 351 | 0.27 (0.14 – 0.54) | 73 (46 to 86) % |
|  | 3 | 7 | 165 | 0.07 (0.02 – 0.20) | 93 (80 to 98) % |
| Vasculitis | 0 | 17 | 44 | 1 |  |
|  | 1 | 57 | 276 | 0.54 (0.24 – 1.26) | 46 (-26 to 76) % |
|  | 2 | 22 | 98 | 0.72 (0.23 – 2.23) | 28 (-123 to 77) % |
|  | 3 | 10 | 40 | 0.90 (0.20 – 4.09) | 10 (-309 to 80) % |
| Psoriasis | 0 | 67 | 336 | 1 | 51 (39 to 60) % |
|  | 1 | 195 | 1,555 | 0.61 (0.42 – 0.89) | 39 (11 to 58) % |
|  | 2 | 52 | 457 | 0.39 (0.22 – 0.69) | 61 (31 to 78) % |
|  | 3 | 24 | 201 | 0.28 (0.13 – 0.60) | 72 (40 to 87) % |
| Atopic dermatitis | 0 | 95 | 478 | 1 |  |
|  | 1 | 318 | 2,405 | 0.56 (0.42 – 0.77) | 44 (23 to 58) % |
|  | 2 | 75 | 713 | 0.27 (0.17 – 0.43) | 73 (57 to 83) % |
|  | 3 | 37 | 315 | 0.27 (0.15 – 0.51) | 73 (49 to 85) % |
| Inflammatory bowel disease | 0 | 95 | 852 | 1 |  |
|  | 1 | 423 | 4,190 | 0.69 (0.53 – 0.91) | 31 (9 to 47) % |
|  | 2 | 98 | 1,003 | 0.42 (0.28 – 0.64) | 58 (36 to 72) % |
|  | 3 | 38 | 468 | 0.24 (0.14 – 0.42) | 76 (58 to 86) % |

^1^ Model 3: Adjusted for age, sex, duration of follow-up, body mass index, Charlson’s comorbidity index, ethnicity, deprivation (Index of Multiple Deprivation quintile), alcohol intake, smoking status, oral corticosteroid prescription in the 90 days prior to the index date, prior SARS-CoV-2 infection and/or COVID‑19 before start of follow-up, healthcare utilisation (general practice consultations and hospital admissions in the previous 12 months, quintiles) and clinical risk group status. aVE (95% CI) was calculated as 1- aOR (95 %CI) from model 3.

**Table S4 Hospitalisation and/or death due to COVID-19 and number of COVID-19 booster vaccinations: subgroup analysis**

| **Category** | **Booster doses** | **Cases**  **n = 2,178** | **Controls**  **n = 17, 750** | **Adjusted OR**  **(95% CI)^1^** | **aVE (95 % CI)^1^** |
| --- | --- | --- | --- | --- | --- |
| **Age group** |  |  |  |  |  |
| Age ≥65 years | 0 | 230 | 881 | 1 |  |
|  | 1 | 752 | 5,526 | 0.36 (0.30 – 0.45) | 64 (55 to 70) % |
|  | 2 | 235 | 2,451 | 0.15 (0.12 – 0.21) | 85 (79 to 88) % |
|  | 3 | 111 | 1,300 | 0.10 (0.07 – 0.15) | 90 (85 to 93) % |
| Age <65 years | 0 | 149 | 1,212 | 1 |  |
|  | 1 | 592 | 5,368 | 0.85 (0.68 – 1.06) | 15 (-6 to 32) % |
|  | 2 | 90 | 796 | 0.68 (0.46 – 0.99) | 32 (1 to 54) % |
|  | 3 | 19 | 216 | 0.42 (0.23 – 0.79) | 58 (21 to 77) % |
| **Sex** |  |  |  |  |  |
| Male | 0 | 148 | 846 | 1 |  |
|  | 1 | 549 | 4,252 | 0.51 (0.40 – 0.65) | 49 (35 to 60) % |
|  | 2 | 118 | 1,297 | 0.20 (0.14 – 0.30) | 80 (70 to 86) % |
|  | 3 | 58 | 613 | 0.17 (0.11 – 0.28) | 83 (72 to 89) % |
| Female | 0 | 231 | 1,247 | 1 |  |
|  | 1 | 795 | 6,642 | 0.58 (0.48 – 0.71) | 42 (29 to 52) % |
|  | 2 | 207 | 1,950 | 0.36 (0.27 – 0.47) | 64 (53 to 73) % |
|  | 3 | 72 | 903 | 0.23 (0.15 – 0.33) | 77 (67 to 85) % |
| **Prior COVID-19** |  |  |  |  |  |
| Yes | 0 | 29 | 170 | 1 |  |
|  | 1 | 33 | 704 | 0.35 (0.17 – 0.73) | 65 (27 to 83) % |
|  | 2 | 7 | 153 | 0.21 (0.06 – 0.75) | 79 (25 to 94) % |
|  | 3 | 1 | 44 | 0.08 (0.01 – 1.04) | 92 (-4 to 99) % |
| No | 0 | 350 | 1,923 | 1 |  |
|  | 1 | 1,311 | 10,190 | 0.58 (0.50 –0.67) | 42 (33 to 50) % |
|  | 2 | 318 | 3,094 | 0.30 (0.24 – 0.37) | 70 (63 to 76) % |
|  | 3 | 129 | 1,472 | 0.21 (0.15 – 0.28) | 79 (72 to 85) % |
| **Immune suppressing drug^2^** | | | | |  |
| Methotrexate | 0 | 127 | 724 | 1 |  |
|  | 1 | 444 | 4,259 | 0.47 (0.36 – 0.61) | 53 (39 to 64) % |
|  | 2 | 113 | 1,479 | 0.24 (0.16 – 0.35) | 76 (65 to 84) % |
|  | 3 | 52 | 730 | 0.18 (0.11 – 0.30) | 82 (70 to 89) % |
| Thiopurine (azathioprine / 6-mercaptopurine) | 0 | 30 | 220 | 1 |  |
|  | 1 | 185 | 1,206 | 1.26 (0.78 – 2.02) | -26(-102 to 22)% |
|  | 2 | 43 | 249 | 1.16 (0.57 – 2.35) | -16(-135 to 43)% |
|  | 3 | 5 | 97 | 0.36 (0.11 – 1.19) | 64 (-19 to 89) % |
| 5-ASA / sulfasalazine | 0 | 124 | 918 | 1 |  |
|  | 1 | 407 | 4,571 | 0.44 (0.34 – 0.57) | 56 (43 to 66) % |
|  | 2 | 107 | 1,275 | 0.22 (0.15 – 0.32) | 78 (68 to 85) % |
|  | 3 | 55 | 575 | 0.17 (0.10 – 0.28) | 83 (72 to 90) % |
| Leflunomide | 0 | 10 | 73 | 1 |  |
|  | 1 | 42 | 370 | 0.59 (0.23 – 1.50) | 41 (-50 to 77) % |
|  | 2 | 14 | 112 | 0.33 (0.09 – 1.25) | 67 (-25 to 91) % |
|  | 3 | 1 | 34 | 0.07 (0.01 – 1.00) | 93 (0 to 99) % |
| Tacrolimus or sirolimus or ciclosporin or mycophenolate | 0 | 23 | 39 | 1 |  |
|  | 1 | 95 | 207 | 0.76 (0.36 – 1.59) | 24 (-59 to 64) % |
|  | 2 | 19 | 76 | 0.63 (0.21 – 1.95) | 37 (-95 to 79) % |
|  | 3 | 3 | 26 | 0.30 (0.05 – 1.84) | 70 (-84 to 95) % |

^1^ Model 3: Adjusted for age, sex, duration of follow-up, body mass index, Charlson’s comorbidity index, ethnicity, deprivation (Index of Multiple Deprivation quintile), alcohol intake, smoking status, oral corticosteroid prescription in the 90 days prior to the index date, prior SARS-CoV-2 infection and/or COVID‑19 before start of follow-up, healthcare utilisation (general practice consultations and hospital admissions in the previous 12 months, quintiles) and clinical risk group status. aVE (95% CI) was calculated as 1- aOR (95 %CI) from model 3. ^2^Within 90 days of the index date.

**Table S5 Conditional logistic regression model examining association between hospitalisation and/or death due to COVID-19 and COVID-19 booster vaccination**

|  | **Cases**  **n = 2,178** | | **Controls**  **n = 17,750** | **Model 1 aOR (95% CI)^1^** | **Model 2 aOR (95% CI)^2^** | **Model 3 aOR (95% CI)^3^** | **aVE**  **(95% CI)^3^** |
| --- | --- | --- | --- | --- | --- | --- | --- |
|  |  | |  |  |  |  |  |
| **Booster doses** |  | |  |  |  |  |  |
| 0 | 379 | | 2,093 | 1 | 1 | 1 |  |
| 1 | 1,344 | | 10,894 | 0.49 (0.42–0.57) | 0.47 (0.40 –0.55) | 0.47 (0.39–0.57) | 53 (43 to 61) % |
| 2 | 325 | | 3,247 | 0.24 (0.20–0.30) | 0.22 (0.18 –0.29) | 0.23 (0.18–0.30) | 77 (70 to 82) % |
| 3 | 130 | | 1,516 | 0.14 (0.11–0.19) | 0.14 (0.11 –0.19) | 0.14 (0.10–0.20) | 86 (80 to 90) % |
| **Booster campaign (start and end dates)** | **Booster** | |  |  |  |  |  |
| Autumn 2021 (20 Sep 2021–20 Mar 2022) | No | 310 | 1,726 | 1 | 1 | 1 |  |
|  | Yes | 940 | 7,801 | 0.36 (0.29–0.43) | 0.33 (0.27 –0.41) | 0.36 (0.28–0.46) | 64 (54 to 72) % |
| Spring 2022 (21 Mar 2022–04 Sep 2022) | No | 626 | 4,440 | 1 | 1 | 1 |  |
|  | Yes | 163 | 1,924 | 0.44 (0.36–0.55) | 0.46 (0.37 –0.56) | 0.49 (0.39–0.62) | 51 (38 to 61) % |
| Autumn 2022 (05 Sep 2022–04 Mar 2023) | No | 252 | 1,207 | 1 | 1 | 1 |  |
|  | Yes | 419 | 3,342 | 0.25 (0.20–0.32) | 0.25 (0.19 –0.33) | 0.24 (0.17–0.34) | 76 (66 to 83) % |
| **Time since last booster** |  | |  |  |  |  |  |
| No booster | 379 | | 2093 | 1 | 1 | 1 |  |
| 0-13 days | 61 | | 1011 | 0.25 (0.18–0.33) | 0.27 (0.20 –0.37) | 0.27 (0.19 –0.38) | 73 (62 to 81) % |
| 14-90 days | 613 | | 6541 | 0.36 (0.31–0.43) | 0.38 (0.31 –0.46) | 0.41 (0.33 –0.50) | 59 (50 to 67) % |
| 91-180 days | 736 | | 5865 | 0.50 (0.42– 0.60) | 0.51 (0.42 –0.62) | 0.52 (0.42 –0.65) | 48 (35 to 58) % |
| 181-270 days | 242 | | 1457 | 0.75 (0.60–0.93) | 0.71 (0.56 – 0.89) | 0.63 (0.49 –0.82) | 37 (18 to 51) % |
| 271-365 days | 80 | | 560 | 0.68 (0.50–0.93) | 0.60 (0.43 – 0.84) | 0.53 (0.36 – 0.78) | 47 (22 to 64) % |
| ≥366 days | 67 | | 223 | 1.55 (1.09–2.20) | 1.03 (0.69 –1.56) | 1.01 (0.62 – 1.63) | -1 (-63 to 38) % |

^1^ Adjusted for age, sex, and duration of follow-up.
^2^ Adjusted for age, sex, duration of follow-up, body mass index, Charlson’s comorbidity index, ethnicity, deprivation (Index of Multiple Deprivation quintile), alcohol intake, smoking status, oral corticosteroid prescription in the 90 days prior to the index date, and prior COVID‑19 before start of follow-up.
^3^ Adjusted as in Model 2 plus healthcare utilisation (general practice consultations and hospital admissions in the previous 12 months, quintiles) and clinical risk group status. aVE (95% CI) was calculated as 1- aOR (95 %CI) from model 3.

**Table S6 Hospitalisation and/or death due to COVID-19 and number of COVID-19 booster vaccinations after excluding those with a primary-care or secondary-care record of SARS-CoV-2 infection or COVID-19 prior to study start**

|  | **Number cases n=2,108** | **Number controls n=16,679** | **Model 1 aOR (95% CI) ^1^** | **Model 2 aOR (95% CI) ^2^** | **Model 3 aOR (95% CI) ^3^** | **aVE (95% CI)^3^** |
| --- | --- | --- | --- | --- | --- | --- |
|  |  |  |  |  |  |  |
| **Booster doses** |  |  |  |  |  |  |
| 0 | 350 | 1,923 | 1 | 1 | 1 |  |
| 1 | 1,311 | 10,190 | 0.57 (0.49–0.65) | 0.57 (0.49–0.65) | 0.58 (0.50–0.67) | 42 (33 to 50) % |
| 2 | 318 | 3,094 | 0.31 (0.25–0.37) | 0.30 (0.24–0.37) | 0.30 (0.24–0.37) | 70 (63 to 76) % |
| 3 | 129 | 1,472 | 0.20 (0.15–0.26) | 0.20 (0.15–0.27) | 0.21 (0.15–0.28) | 79 (72 to 85) % |

^1^ Adjusted for age, sex, and duration of follow-up.
^2^ Adjusted for age, sex, duration of follow-up, body mass index, Charlson’s comorbidity index, ethnicity, deprivation (Index of Multiple Deprivation quintile), alcohol intake, smoking status, oral corticosteroid prescription in the 90 days prior to the index date, and prior COVID‑19 before start of follow-up.
^3^ Adjusted as in Model 2 plus healthcare utilisation (general practice consultations and hospital admissions in the previous 12 months, quintiles) and clinical risk group status. aVE (95% CI) was calculated as 1- aOR (95 %CI) from model 3.

**Table S7 Hospitalisation and/or death due to COVID-19 and number of COVID-19 booster vaccinations excluding outcomes with a positive test result alone**

|  | **Number cases n=701** | **Number controls n=17,750** | **Model 1 aOR (95% CI) ^1^** | **Model 2 aOR (95% CI) ^2^** | **Model 3 aOR (95% CI) ^3^** | **aVE (95% CI)^3^** |
| --- | --- | --- | --- | --- | --- | --- |
|  | 701 | 17,750 |  |  |  |  |
| **Booster doses** |  |  |  |  |  |  |
| 0 | 209 | 2,093 | 1 | 1 | 1 |  |
| 1 | 380 | 10,894 | 0.30 (0.25–0.37) | 0.31 (0.25–0.38) | 0.30 (0.24–0.37) | 70 (63 to 76) % |
| 2 | 74 | 3,247 | 0.15 (0.10–0.21) | 0.15 (0.11–0.21) | 0.14 (0.09–0.20) | 86 (80 to 91) % |
| 3 | 38 | 1,516 | 0.14 (0.09–0.22) | 0.15 (0.09–0.23) | 0.13 (0.08–0.22) | 87 (78 to 92) % |

^1^ Adjusted for age, sex, and duration of follow-up.
^2^ Adjusted for age, sex, duration of follow-up, body mass index, Charlson’s comorbidity index, ethnicity, deprivation (Index of Multiple Deprivation quintile), alcohol intake, smoking status, oral corticosteroid prescription in the 90 days prior to the index date, and prior COVID‑19 before start of follow-up.
^3^ Adjusted as in Model 2 plus healthcare utilisation (general practice consultations and hospital admissions in the previous 12 months, quintiles) and clinical risk group status. aVE (95% CI) was calculated as 1- aOR (95 %CI) from model 3.

**Table S8** **Hospitalisation and/or death due to COVID-19 and number of COVID-19 booster vaccinations in patients who received a two-dose primary course of COVID-19 vaccination**

|  | **Number cases n=2,097** | **Number controls n-=17,193** | **Model 1 aOR (95% CI) ^1^** | **Model 2 aOR (95% CI) ^2^** | **Model 3 aOR (95% CI) ^3^** | **aVE (95% CI)^3^** |
| --- | --- | --- | --- | --- | --- | --- |
|  |  |  |  |  |  |  |
| **Booster doses** |  |  |  |  |  |  |
| 0 | 355 | 1,992 | 1 | 1 | 1 |  |
| 1 | 1,302 | 10,566 | 0.55 (0.49–0.64) | 0.55 (0.48–0.63) | 0.56 (0.48–0.65) | 44 (35 to 52) % |
| 2 | 312 | 3,153 | 0.31 (0.25–0.37) | 0.29 (0.24–0.36) | 0.29 (0.23–0.37) | 71 (63 to 77) % |
| 3 | 128 | 1,482 | 0.21 (0.16–0.27) | 0.20 (0.15–0.27) | 0.21 (0.16–0.29) | 79 (71 to 84) % |

^1^ Adjusted for age, sex, and duration of follow-up.
^2^ Adjusted for age, sex, duration of follow-up, body mass index, Charlson’s comorbidity index, ethnicity, deprivation (Index of Multiple Deprivation quintile), alcohol intake, smoking status, oral corticosteroid prescription in the 90 days prior to the index date, and prior COVID‑19 before start of follow-up.
^3^ Adjusted as in Model 2 plus healthcare utilisation (general practice consultations and hospital admissions in the previous 12 months, quintiles) and clinical risk group status. aVE (95% CI) was calculated as 1- aOR (95 %CI) from model 3.


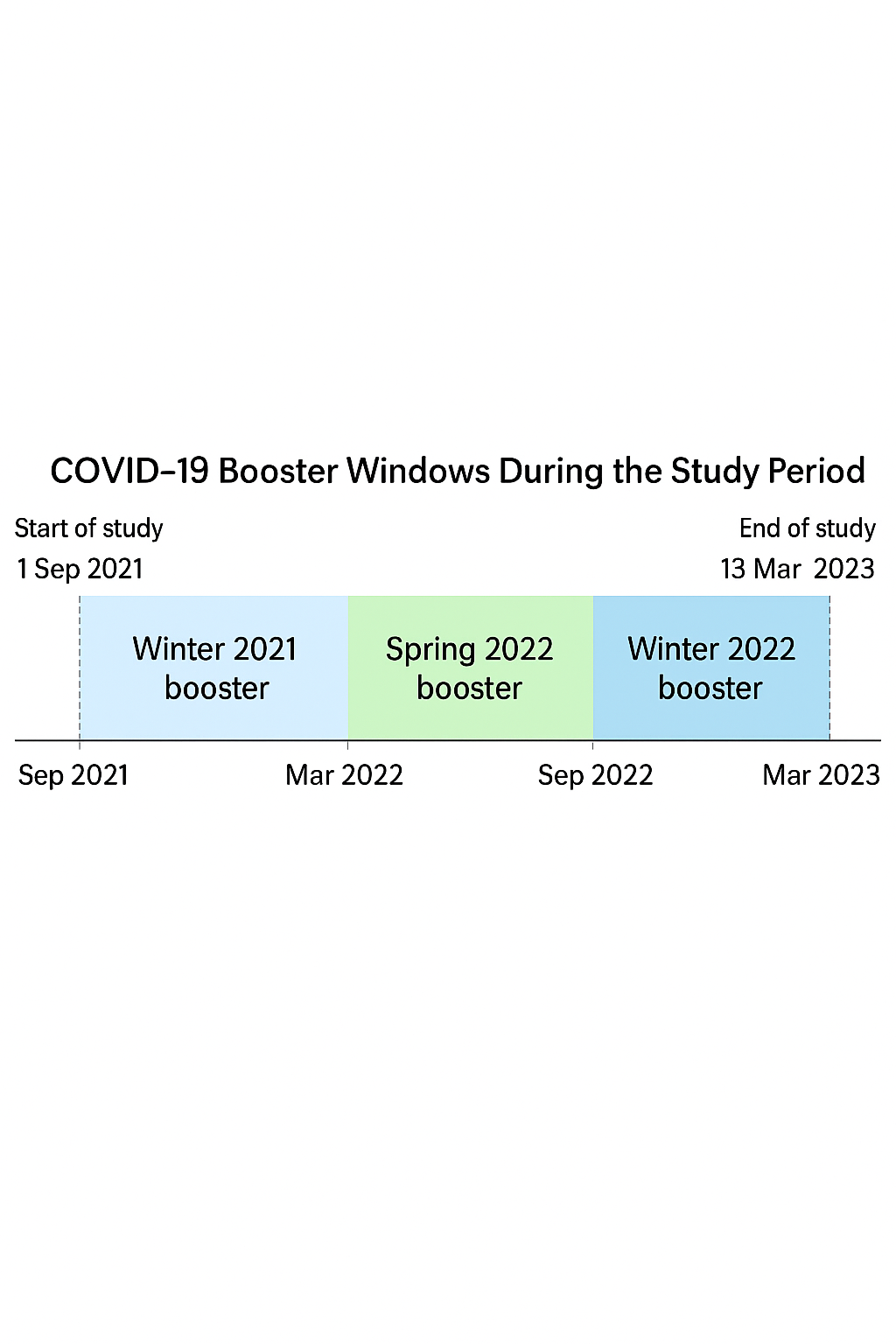


16

**Figure S1: COVID-19 Booster windows as defined within study period**
